# Specific nasopharyngeal *Corynebacterium* strains serve as gatekeepers against SARS-CoV-2 infection

**DOI:** 10.1007/s11357-023-00850-1

**Published:** 2023-06-20

**Authors:** Dora Szabo, Eszter Ostorhazi, Balazs Stercz, Nora Makra, Kinga Penzes, Katalin Kristof, Istvan Antal, Janos M. Rethelyi, Reka I. Zsigmond, Ede Birtalan, Bela Merkely, Laszlo Tamas

**Affiliations:** 1 Institute of Medical Microbiology, Semmelweis University, Üllői Street 26, 1085 Budapest, Hungary; 2 Human Microbiota Study Group, Semmelweis University-Eötvös Lóránd Research Network, Budapest, Hungary; 3 Institute of Laboratory Medicine, Clinical Microbiology Laboratory, Semmelweis University, Budapest, Hungary; 4 Department of Pharmaceutics, Semmelweis University, Budapest, Hungary; 5 Department of Psychiatry and Psychotherapy, Semmelweis University, Budapest, Hungary; 6 Department of Oto-Rhino-Laryngology and Head and Neck Surgery, Semmelweis University, Budapest, Hungary; 7 Heart and Vascular Center, Semmelweis University, Budapest, Hungary; 8 Department of Voice, Speech and Swallowing Therapy, Faculty of Health Sciences, Semmelweis University, Budapest, Hungary

**Keywords:** SARS-CoV-2, Nasopharyngeal microbiome, *Corynebacterium accolens*, ACE-2, TMPRSS2, Cathepsin L, TAG lipase S1

## Abstract

The SARS-CoV-2 virus is still causing a worldwide problem. The virus settles primarily on the nasal mucosa, and the infection and its course depend on individual susceptibility. Our aim was to investigate the nasopharynx composition’s role in the individual susceptibility. During the first phase of SARS-CoV-2 pandemic, nasopharyngeal microbiome samples of close contact unvaccinated patients were investigated by 16S rRNA analysis and by culturing. The whole genome of cultured *Corynebacteria* was sequenced. The relative expression of ACE2, TMPRSS2, and cathepsin L on Caco-2 cells and the strength of S1-ACE2 binding were determined in the presence of *Corynebacteria*. From 55 close contacts exposed to identical SARS-CoV-2 exposure, 26 patients became infected and 29 remained uninfected. The nasopharyngeal microbiome analysis showed significantly higher abundance of *Corynebacteria* in uninfected group. *Corynebacterium accolens* could be cultivated only from uninfected individuals and* Corynebacterium propinquum* from both infected and uninfected. *Corynebacteria* from uninfected patient significantly reduced the ACE2 and cathepsin L expression. *C. accolens* significantly reduced the TMPRSS2 expression compared to other *Corynebacteria*. Furthermore, *Corynebacterium *spp. weakened the binding of the S1-ACE2. Most *C. accolens* isolates harbored the TAG lipase LipS1 gene. Based on these results, the presence of *Corynebacterium* spp. in the nasopharyngeal microbiota, especially *C. accolens* strains, could reduce the individual susceptibility to SARS-CoV-2 infection by several mechanisms: by downregulation the ACE2, the TMPRSS2 receptors, and cathepsin L in the host; through the inhibition of S1-ACE2 binding; and lipase production. These results suggest the use of *C. accolens* strains as probiotics in the nasopharynx in the future.

## Introduction

Epithelial cells of the upper and lower respiratory tracts are primary targets for airborne infections including SARS-CoV-2. These cells are covered by complex bacterial communities mainly in the upper respiratory tract that may directly or indirectly interact with coronaviruses. The commensal microbiota may also prevent infection by regulating innate and adaptive host immune responses [1]. A relationship was observed between the composition of nasopharyngeal microbiota and individual susceptibility to upper respiratory viral (e.g., influenza) and bacterial infections [2–4].

The COVID-19 epidemic is still a serious problem. One of the reasons for this is that the evolution of SARS-CoV-2 has led to the emergence of several new variants. The new variants show distinct cell tropism and mode of entry compared to other SARS-CoV-2 variants. Unlike the original, other SARS-CoV-2 variants can primarily use the cathepsin L entry route in addition to the ACE2 and TMPRSS2 cell entry route [5, 6].

The aim of our study was to investigate the nasopharyngeal microbiota of SARS-CoV-2 infected and close-contact non-infected individuals exposed to identical external environment between May and June 2020, and in November–December 2020 at Semmelweis University. During this period, all subjects in the study were still unvaccinated.

## Methods

### Ethical approval and involvement of patients in the study, consent to participate

Sample collection protocols were approved by the Ethics Committee of Semmelweis University (SE RKEB: 217/2020) and by National Public Health Center (38215-6/2020 EÜIG). The present study was conducted in accordance with ethical standards that promote and ensure respect and integrity for all human subjects and the Declaration of Helsinki. We state that no sex-based or race/ethnicity-based differences were present. All research was performed in accordance with guideline and regulation of Semmelweis University. Written informed consent has been obtained from the participants (or their legal guardians) of the study before sampling.

In the periods between May and June 2020 in a closed community during a local epidemic of SARS-CoV-2 and between November and December 2020, longitudinal household transmissions were analyzed at Semmelweis University, in 2 waves of the COVID-19 epidemic in Hungary. Nasopharyngeal swab samples were collected from 27 inpatients and 28 outpatients, respectively. Of the subjects included in the study, 26 had symptomatic and PCR-confirmed SARS-CoV-2 infection. The selection criteria for the negative control group (29 persons) were close contact with infected subjects, multiple PCR-negative results during the study period, no symptoms, no vaccination, and no previous evidence of COVID-19 infection verified with negative AdviseDx SARS-CoV-2 IgG II CMIA test (Abbott, USA). A patient was considered negative if the person had a negative PCR and serological test. A patient was considered positive with a positive PCR test. Patients with positive serological test, but negative PCR test were ruled out of the study.

### Culture methods

Nasopharyngeal swab samples of the second study group patients were inoculated on Columbia blood agar (Biolab, Hungary). Antimicrobial susceptibility disk containing 50 µg fosfomycin (Oxoid, Sweden) was used to select *Corynebacterium* strains from other bacterial participants. All cultured bacteria were identified by MALDI-TOF (Bruker Daltonik, Germany).

For the detection of bacterial products that affect the ACE2-spike binding, 24 different *Corynebacterium* strains previously isolated from the second study group patients’ nasopharyngeal swabs were cultured in EMEM medium (Lonza Bioscience, USA) at 37 °C in an atmosphere containing 5% CO_2_.

The human colonic epithelial cell line Caco-2 (Sigma-Aldrich, USA) was cultured in EMEM medium at 37 °C in a humidified atmosphere containing 5% CO_2_ and the medium was changed every 2 days. No antibiotics were added to allow the co-cultivation of bacteria. The Caco-2 cells were cultivated alone for 4 days before the addition of the overnight cultures of *Corynebacteria*. Human Caco-2 cells and bacteria were co-cultured for additional 24 h.

We examined how each *Corynebacterium* strain and the two *Streptococcus* strains—one *Streptococcus mitis* and one *Streptococcus sanguis*—mutually influence reproduction. A pure bacterial culture from each bacterial strain was suspended in saline, its turbidity was standardized at 0.5 McFarland, and it was swabbed uniformly across Columbia blood agar plates forming a basic lawn. All *Corynebacterium* strain lines were grafted onto both *Streptococcus* lawns and, conversely, the two *Streptococcus* strains were grafted onto each *Corynebacterium* lawn. The inhibitory effect of lawns on the growth of the lines was investigated.

### DNA isolation, 16S rRNA gene library preparation, and MiSeq sequencing

DNA isolation was performed according to manufacturer’s protocol by the ZymoBIOMICS DNA Miniprep Kit (Zymo Research Corp, USA). Isolated DNA samples were placed at − 80 °C until PCR amplification. Concentration of genomic DNA was measured using a Qubit2.0 Fluorometer with Qubit dsDNA HS Assay Kit (Thermo Fisher Scientific, USA). Bacterial DNA was amplified with tagged primers covering the V3-V4 region of bacterial 16S rRNA gene. PCR and DNA purification were performed according to Illumina’s protocol. PCR product libraries were assessed using the DNA 1000 Kit with Agilent 2100 Bioanalyzer (Agilent Technologies, Germany). Equimolar concentrations of libraries were pooled and sequenced on the Illumina MiSeq platform (Illumina, USA), using MiSeq Reagent Kit v3 (600 cycles PE).

In order to evaluate the contribution of extraneous DNA from reagents, extraction negative controls and PCR negative controls were included in every run. To ensure reproducibility, each sample was independently extracted and sequenced twice.

Raw sequencing data were retrieved from the Illumina BaseSpace and uploaded to the CosmosID Bioinformatics Platform for evaluation (CosmosID Metagenomics Cloud, app.cosmosid.com, CosmosID Inc., www.cosmosid.com).

### Whole genome sequencing

From 22 *Corynebacterium* strains, genomic DNA were isolated using the NucleoSpin Microbial DNA Kit (Macherey Nagel, Germany). The NGS libraries in both cases were prepared using the Nextera DNA Flex Library Prep Kit according to the manufacturer’s instructions (Illumina, USA). The libraries were paired-end sequenced on a MiSeq instrument using 250 bp read length (MiSeq Reagent Kit v2, 500-cycles). The fastq files were used to assemble the de novo draft genome sequence using the SPAdes de novo genome assembler (version 3.7.1) and the BioNumerics version 7.6 software (Applied Maths NV, Belgium).

### ACE2-spike binding inhibition

Twenty-four broth cultures of different bacterial strains were centrifuged at 8000 g for 3 min and the cell free supernatants were tested with the Cayman Chemical (Cayman Chemical Company, USA) SARS-CoV-2 spike-ACE2 interaction screening assay kit, according to the manufacturer’s instructions. Briefly, the assay uses a recombinant rabbit Fc-tagged SARS-CoV-2 spike S1 RBD attached to the plate precoated with a mouse anti-rabbit antibody. Fifty microliters of supernatant was used to test competition of RBD-ACE2 interaction. The test uses recombinant His-tagged ACE2 protein, HRP conjugated anti-His antibody, and TMB substrate. Results were read at 450-nm wavelength. All samples were assayed in triplicate. The mean OD of the inhibition control reagent included in the kit was considered to induce 0% binding activity, and the mean OD of sterile broth medium was considered as 100% of the initial activity.

### Examination of ACE2, TMPRSS2, and cathepsin L expression of Caco-2 cells after bacterial co-culture

Caco-2 cells were cultivated for 4 days, an overnight culture of *Corynebacteria* was added to the Caco-2 cell lines, and for 24 h were further co-cultivated. The cells were washed by PBS and 0.25% trypsin and centrifuged. The total RNA was isolated by innuPREP RNA Mini Kit 2.0 (Analytik Jena GmbH, Germany) according to manufacturer’s instructions. RNA concentrations were determined using a NanoDrop 1000 spectrophotometer (Thermo Fisher Scientific, USA). Ten to 100 ng of RNA was used for RT-PCR assay performed using the PrimeScript RT reagent kit (Takara Bio, USA) and amplified the resulting cDNA on a qTOWER 3G (Analytik Jena GmbH, Germany) instrument in the presence of selected primers.

The primers for ACE2 were 5′-GGG ATC AGA GAT CGG AAG AAG AAA-3′ forward and 5′-AGG AGG TCT GAA CAT CAT CAG TG-3′ reverse. The primers for TMPRSS2 were 5′-AAT CGG TGT GTT CGC CTC TAC-3′ forward and 5′-CGT AGT TCT CGT TCC AGT CGT-3′ reverse. The primers for cathepsin L were 5′-CTG GTG GTT GGC TAC GGA TT-3′, CTSL forward and 5′-CTC CGG TCT TTG GCC ATC TT-3′ reverse. The primers for GAPDH were 5′-CTA CTG GCG CTG GCA AGG CTG T-3′ forward and 5′-GCC ATG AGG TCC ACC ACC CTG CTG-3′ reverse.

Relative mRNA expression was calculated by means of the change in cycle threshold (ΔΔCt) and normalized to the geometric mean of housekeeping genes GAPDH. Basal mRNA levels of ACE2 and TMPRSS2 were compared with those of housekeeping gene GAPDH by calculating the difference between their Ct. All experiments were performed four times.

### Data availability

The metagenomics datasets generated during the current study are deposited in the NCBI Short Read Archive (SRA) (https://www.ncbi.nlm.nih.gov/sra) under accession numbers PRJNA728384 and PRJNA842591. The WGS of the selected *Corynebacteria* spp. are available in the PRJNA842591 Bioproject under Biosample SAMN28690426–SAMN28690445 numbers.

### Statistical analysis

Patient groups were compared with a two-sided *t*-test for independent groups in case of age and were compared with Mann-Whitney *U* test in case of gender, smoking status, underlying diseases, and previous antibiotic treatment.

The significance of the difference between relative abundance of taxon levels was calculated with the Mann-Whitney-Wilcoxon test. For analyzing the difference between the OD levels in ACE2-spike binding inhibition ELISA test, two-tailed Student’s *t*-test was used. In ACE2 and TMPRSS2 expression studies, the difference between mRNA levels measured in the different bacterial co-culture groups was calculated by two-tailed Student’s *t*-test. Statistical significance between cohorts was implemented using CHAO1, Simpson, and Shannon index for microbiome alpha diversity and Jaccard or by Bay-Curtis test for beta diversity.

## Results

### Patients involved in the study, description of the virus exposition

The first study was completed between May and June 2020 in a closed community during a local epidemic of SARS-CoV-2 at the Department of Psychiatry and Psychotherapy at Semmelweis University. A patient admitted for acute psychiatric illness was found to be COVID positive after admission to the psychiatric ward. Given that the psychiatric patients in this closed community were not suitable for contact isolation due to their mental illness, they acquired the infection from the index COVID patient or from their already infected fellow patients. Despite this, of the 27 patients in close contact with each other in the secure psychiatric ward, 16 patients became infected with the SARS-CoV-2 virus (13 men and 3 women), and the remaining 11 patients were not infected with the virus. During the observed 1.5-month period, the average number of PCR tests performed per patient was 13, with a minimum of 7 and a maximum of 18 SARS-CoV-2 PCR tests per patient from the nasopharyngeal samples.

The second study analyzed longitudinal household transmissions between November and December 2020 at Semmelweis University. In these cases, a member of the family was forced to self-quarantine due to their COVID-positivity along with family members living in the same household. Family members shared a common dining room, bedroom, and restroom. Several of the people living in joint quarantine were infected with the SARS-CoV-2 virus, but some were not. Ten SARS-CoV-2 positive individuals and their family members, eighteen individuals were quarantined together.

Altogether, from the total 55 persons being close contacts, 26 patients became infected, while 29 persons remained uninfected despite identical virus exposure.

A total of 55 patients were enrolled in the study. Baseline demographics were overall well balanced, the mean age was 44 years, 58% of the study population were male, 62% of patients were non-smokers, 29% of the patients had underlying diseases, and 11% received antibiotic treatment in the last 3 months. However, significant difference was observed in terms of gender; significantly (*p* < 0.001) more men were infected with the SARS-CoV-2 virus. However, there were no significant differences observed in the other parameters: age, smoking, underlying diseases, previous antibiotic treatment among the patients in the SARS-CoV-2 positive and SARS-CoV-2 negative groups. The baseline characteristics—age, gender, smoking, underlying diseases, and previous antibiotic treatment—of the patients involved in the studies are summarized in Table 1.

**Table 1:** Baseline characteristics of the patients involved the study

Variables *n* (%) or median (IDR)	All patients (*n* = 55)	SARS-CoV-2 positive (*n* = 26)	SARS-CoV-2 negative (*n* = 29)	*p* values
Age (years)	44 (18–88)	41.5 (23–65)	44 (18–88)	*p* = 0.41
Gender, male	32 (58%)	21 (38%)	11 (20%)	*p* = 0.00048***
Smokers	21 (38%)	9 (34%)	9 (31%)	*p* = 0.38
Underlying disease (%)	16 (29%)	7 (27%)	9 (31%)	*p* = 0.96
Diabetes	0%	0%	0%	
Hypertonia	9 (16%)	4 (15%)	5 (17%)	
COPD	3 (5%)	2 (8%)	1 (3%)	
Allergic rhinitis	1 (2%)	0%	1 (3%)	
Asthma	1 (2%)	0%	1 (3%)	
Hypothyreosis	2 (4%)	0%	2 (6%)	
Fibroadenoma mammae	1 (2%)	0%	1 (3%)	
Epilepsy	1 (2%)	0%	1 (3%)	
Chronic lymphoid leukemia	1 (2%)	1 (4%)	0%	
Primer biliary cholangitis	1 (2%)	0%	1 (3%)	
Hashimoto thyroiditis	1 (2%)	0%	1 (3%)	
Arthritis urica	1 (2%)	0%	1 (3%)	
Gilbert syndrome	1 (2%)	1 (4%)	0%	
Strictura oesophagi	1 (2%)	1 (4%)	0%	
Previous antibiotic treatment (< 3 months)	6 (11%)	3 (12%)	3 (10%)	*p* = 0.84
Amoxicillin/clavulanic acid	1 (2%)	1 (4%)	0%	
Ceftriaxone	2 (4%)	0%	2 (7%)	
Cefuroxime	1 (2%)	0%	1 (3%)	
Clarithromycin	1 (2%)	0%	1 (3%)	
Azithromycin	1 (2%)	1 (4%)	0%	
Ciprofloxacin	1 (2%)	0%	1 (3%)	
Piperacillin/tazobactam	1 (2%)	1 (4%*)*	0%	

*p* values were considered significant with ***p < 0.001

### Nasopharyngeal microbiome analysis

**Fig. 1:**
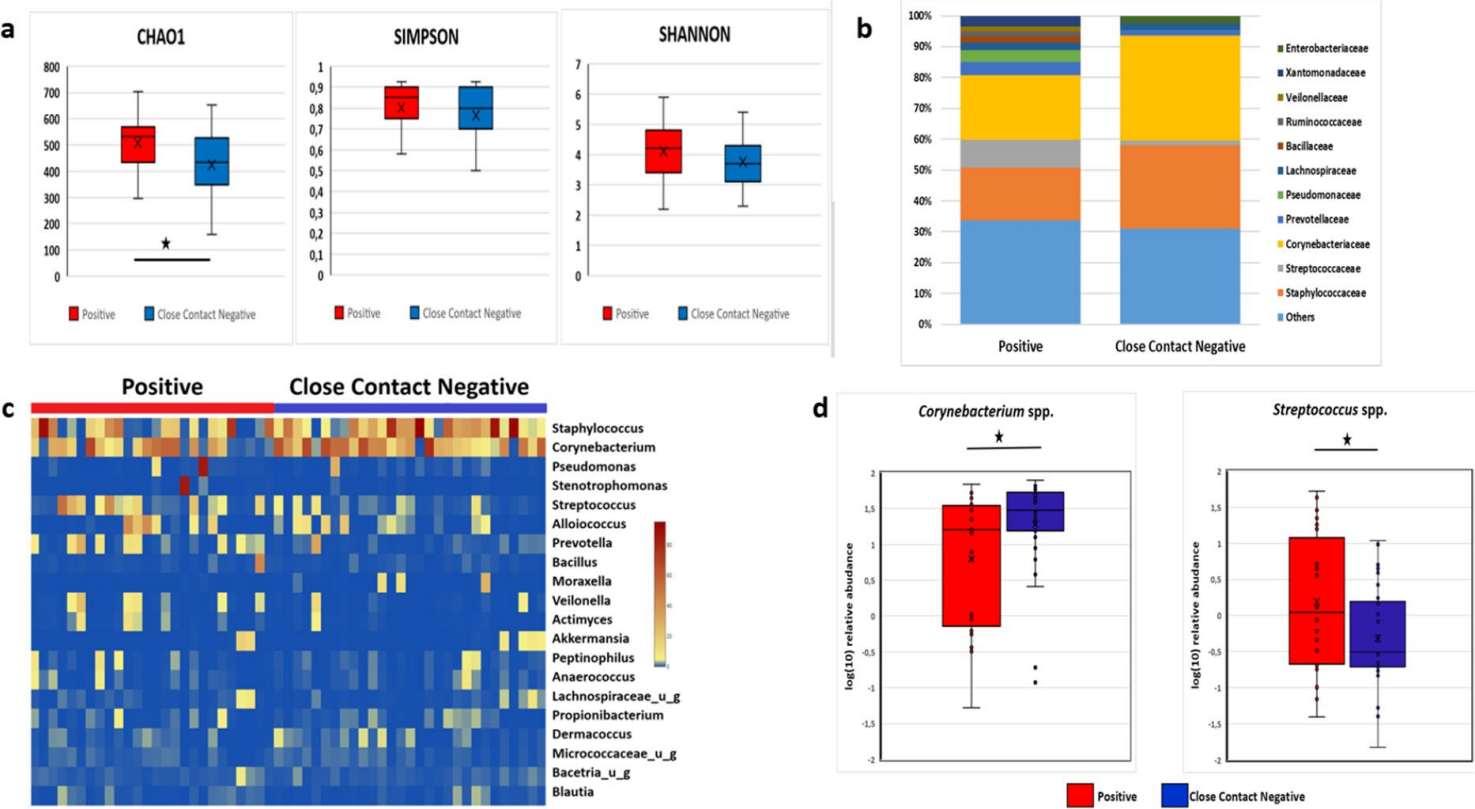
The composition of nasopharyngeal microbiome of SARS-CoV-2 positive patients (red) and the close contact negative persons (blue) analyzed by 16S rRNA analysis. **a** The alpha diversity of the nasopharyngeal microbiome calculated by CHAO1, Simpson, and Shannon index. *p* values were considered significant with *p < 0.05. **b** The most abundant taxa at family level in the nasopharyngeal microbiome of SARS-CoV-2 positive patients (red) and the close contact negative persons (blue). **c** The genus level heatmap of the individuals of SARS-CoV-2 positive patients (red) and the close contact negative persons (blue). **d** The abundance of *Corynebacterium* spp. and *Streptococcus* spp. in the nasopharyngeal microbiome of SARS-CoV-2 positive patients (red) and the close contact negative persons (blue). *p* values were considered significant with *p < 0.05

The overall nasopharyngeal microbiota composition was different between the 26 SARS-CoV-2 positive and 29 SARS-CoV-2 close contact negative subjects in alpha diversity. The alpha diversity indexes were higher in positive patients and lower in negative close contact persons with significant difference in alpha diversity by CHAO1 (*p* = 0.031) 535 [576–455] vs 429 [528–344] (Fig. 1). There were no significant differences in alpha diversity calculated by the Simpson index (*p* = 0.125) 0.8 [0.9–0.7] vs 0.85 [0.9–0.75] and the Shannon index (*p* = 0.206) 4.29 [3.4–4.8] vs 3.7 [3.1–4.3] (Fig. 1a). The beta diversity analysis showed no significant clustering either by Jaccard or by Bay-Curtis analysis.

The taxonomic analysis of the nasopharyngeal microbiome by 16S rRNA showed at phylum level higher amounts of Firmicutes (mean 40.75% vs 38.32%) and marked less Actinobacteria (mean 28.99% vs 40.81%) in SARS-CoV-2 positive patients with no significant differences. At family level and in SARS-CoV-2 negative individuals, the abundance of *Corynebacteriaceae *and *Streptococcaceae *was significantly different (*p* < 0.05) (Fig. 1b). The heatmap of the individual nasopharyngeal microbiome at genus level showed *Staphylococcus*, *Corynebacterium*, and *Streptococcus* dominated microbiome (Fig. 1c). At the genus level, the abundance of *Corynebacterium* spp. was significantly increased (mean value 34.82% vs 20.82%, *p* < 0.05), and the abundance of *Streptococcus* spp. significantly decreased (mean 8.63% vs 1.63%, *p* < 0.05) in the close contact negative uninfected group compared to positive infected group (Fig. 1d). No significant difference was observed regarding the abundance of *Staphylococci*.

### Nasopharyngeal culture analysis

Nasopharyngeal samples of 10 SARS-CoV-2 positive patients and 18 negative close contact family members were conventionally cultured during the second study period.

The positive patients exhibited in four cases *Corynebacterium propinquum*, and in all the cases one or more different *Streptococcus* spp. (*S. mitis*, *S. sanquis*, *S. salivarus*, *S. pneumoniae*, *S. pseudopneumoniae*, *S. oralis*, *S. agalactiae*) strains. Additionally, in four cases *Staphylococcus epidermidis* and in three cases *Moraxella nonliquefaciens*, *Citrobacter koseri*, and* Klebsiella pneumoniae* were cultured.

In contrast, either pure culture of *Corynebacterium*, *Corynebacterium tuberculostearicum*, and *Corynebacterium pseudodiphtericum* were cultivated from the nasopharyngeal samples of SARS-CoV-2 negative close contacts, or these bacteria have been associated with *C. propinquum* or other bacteria including *Staphylococcus aureus* and *S. epidermidis*.

Altogether from the negative close contact uninfected individuals, nine *C. accolens*, one *C. tuberculostearicum*, one *C. pseudodiphtericum*, and seven *C. propinquum* strains were isolated. From the nasopharyngeal samples of SARS-CoV-2 positive patients, only four *C. propinquum* strains were observed (Table 2).

**Table 2:** Characteristics of the isolated *Corynebacterium* spp

	CA1	CA2	CA3	CA4	CA5	CA6	CA7	CA8	CA9	CT	CPT	CP1	CP2	CP3	CP4	CP5	CP6	CP7	CP8	CP9	CP10	CP11
Patient SARS-CoV-2	neg	neg	neg	neg	neg	neg	neg	neg	neg	neg	neg	neg	neg	neg	neg	neg	neg	neg	pos	pos	pos	pos
TAG lipase LipS1	** + **	** + **	** + **	** + **	** − **	** + **	** + **	** + **	** + **	** − **	** − **	** − **	** − **	** − **	** − **	** − **	** − **	** − **	** − **	** − **	** − **	** − **
Growth on *S. mitis/S. sanguis* lawn	** + / + **	** + / + **	** − / + **	** + / + **	** + / + **	** + / + **	** − / − **	** − / + **	** + / + **	** + / + **	** + / + **	** + / + **	** + / + **	** + / + **	** + / + **	** + / + **	** + / + **	** + / + **	** + / + **	** + / + **	** − / + **	** + / + **
Inhibition the growth of *S. mitis/S. sanguis*	** + / − **	** + / + **	** − / − **	** − / − **	** − / − **	** − / − **	** − / − **	** − / − **	** − / − **	** + / + **	** − / − **	** − / − **	** − / − **	** − / − **	** − / − **	** − / − **	** − / − **	** − / − **	** − / − **	** − / − **	** − / − **	** − / − **

*CA Corynebacterium accolens*, *CT Corynebacterium tuberculostearicum*, *CPT Corynebacterium pseudotuberculosis*, *CP Corynebacterium propinquum*

### Whole-genome sequence analysis of the *Corynebacteria*

All the 22 *Corynebacterium* spp. were sequenced. The TAG lipase LipS1 production in eight of nine *C. accolens* strains was confirmed by screening the whole-genome sequence. None of the other *Corynebacterium* spp. harbored the lipase gene (Table 2).

### Mutual cross influence of *Corynebacterium* spp. and *Streptococcus* spp. on each other growth

All isolated *Corynebacteria*’s growth were tested in the presence of one-one randomly selected *S. mitis* and *S. sanguis* strains from infected patients. All 22 *Corynebacterium* spp. except one *C. accolens* were able to grow in the presence of *S. sanguis*; however, the presence of *S. mitis* inhibited the growth of three *C. accolens* strains and one *C. propinquum* strain. Some strains of *Corynebacteria* could inhibit the growth of *S. mitis* (3 of 22) and *S. sanguis* (2 of 22) indicating a competition in mixed culture settings (Table 2).

### The effect of *Corynebacterium* spp. on the ACE2, TMPRSS2, and cathepsin L expression in human cells

Subsequently, nine *C. accolens*, one *C. tuberculostearicum*, one *C. pseudodiphtericum*, and seven *C. propinquum *strains isolated from negative close contacts were further examined. Additionally, four *C. propinquum* strains from positive patients were enrolled in a comprehensive in vitro study*.*

**Fig. 2:**
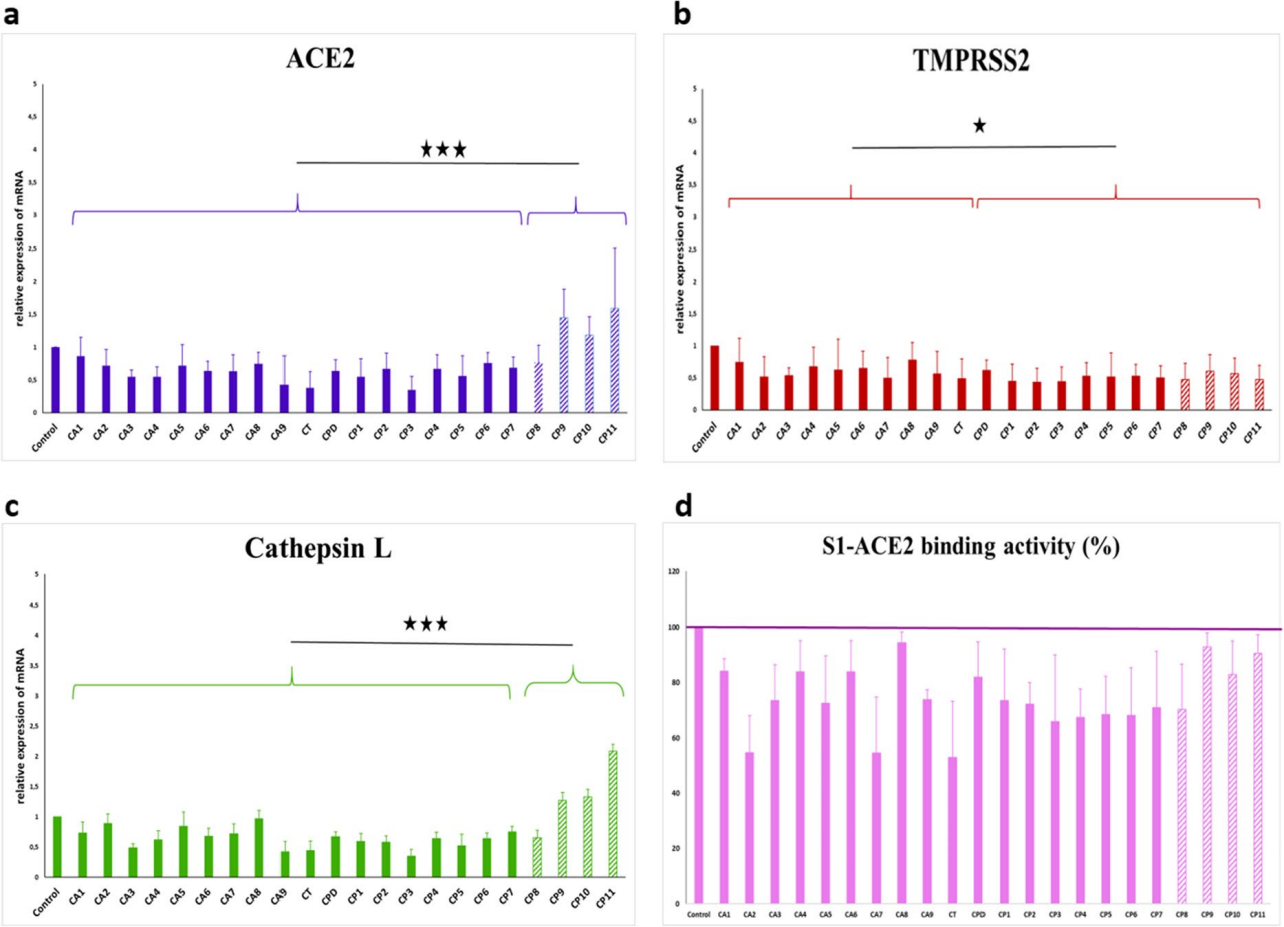
The effect of *Corynebacterium* spp. on the ACE2, TMPRSS2, and cathepsin L expression in human cells and the on the binding strength of S1 protein and ACE2. **a** The relative mRNA expression of ACE2 receptors in the Caco2 cells after 24-h co-culture with *Corynebacteria* spp. (blue bars represent *Corynebacteria* from close contact negative persons and stricked bars represent from SARS-CoV-2 positive patients). *p* values were considered significant with ***p < 0.001. **b** The relative mRNA expression of TMPRSS2 receptors in the Caco2 cells after 24-h co-culture with *Corynebacteria* spp. (red bars represent *Corynebacteria* from close contact negative persons and stricked bars from SARS-CoV-2 positive patients). *p* values were considered significant with *p < 0.05. **c** The relative mRNA expression of cathepsin L receptors in the Caco2 cells after 24-h co-culture with *Corynebacteria* spp. (green bars represent *Corynebacteria* from close contact negative persons and stricked bars from SARS-CoV-2 positive patients). *p* values were considered significant with ***p < 0.001. **d** The relative ACE-2 binding activity of supernatants of *Corynebacteria* spp. (purple bars represent *Corynebacteria* from close contact negative persons and stricked bars from SARS-CoV-2 positive patients). The purple line indicates the control, 100%, where no inhibition is observed. The columns below the purple line indicate a reduced level of the connection between S1 and ACE2

Significant difference (p < 0.001) was observed in ACE2 receptor downregulation among Corynebacterium strains isolated from close contact negative patients and the Corynebacteria isolated from SARS-CoV-2 infected positive patients (Fig. 2a). However, no significant difference was observed between the accolens group (C. accolens and C. tuberculostearicum) and the propinquum group (C. propinquum and C. pseudodiphtericum) in reducing ACE2 relative expression.

All *Corynebacterium* spp. decreased the relative expression of TMPRSS2 showing significant difference (*p* < 0.05) between the accolens group—*C. accolens* and *C. tuberculostearicum*—and propinquum group—*C. pseudotuberculosis* and *C. propinquum* (Fig. 2b). However, no significant differences were observed between the *Corynebacteria* isolated from SARS-CoV-2 positive or close contact negative patients.

There were significant differences (*p* < 0.01) observed in cathepsin L downregulation between *Corynebacteria* isolated from close contact negative patients and the cathepsin L expression-reducing effect of *Corynebacteria* isolated from SARS-CoV-2 positive patients (Fig. 2c). But there were no significant differences between the accolens group—*C. accolens* and *C. tuberculostearicum—*and propinquum group—*C.* *pseudotuberculosis* and *C. propinquum*.

### Effects of isolated *Corynebacterium* spp. on the binding strength of S1 protein and ACE2

By examining the supernatants of all isolated *Corynebacterium* spp. for the strength of S1 protein ACE2 binding, we observed that all *Corynebacterium* spp. strains weakened the binding of S1 protein to the ACE2 receptor. However, there was no significant differences observed neither between the positive and close contact infected patients groups, nor between the accolens and propinquum groups (Fig. 2d).

## Discussion

The correlation of the healthy state of the nasopharyngeal commensal microbiome and infections by SARS-CoV-2 viruses has also been raised recently in several studies, but it has not been clarified until now [7–11].

Even some authors found that SARS-CoV-2 infection does not significantly alter the composition of the microbiome [12], others reported based on 16S rRNA analysis that some *Corynebacteria* strains were more abundant in SARS-CoV-2 negative subjects, and others were more abundant in those infected with the virus [13]. Shilts et al. found that the relative abundance of an unclassified *Corynebacterium* consistently decreased as COVID-19 severity increased [14]. By metagenomic next-generation study—capable to species based identification in contrast to 16S rRNA analysis—Kumpitsch et al. [6] reported a statistically significant decrease of incidence of a commensal organism,* C. accolens*, in the samples of COVID-19-positive patients.

Our study also confirmed that close contact uninfected individuals showed significantly higher levels of *Corynebacterium* genus based on 16S rRNA analysis and in addition less abundant *Streptococcus* genus in their nasopharyngeal microbiome. By conventional culturing, *C. accolens* species often appeared in pure culture of in SARSCoV-2 negative close contacts, but was not cultured from any SARS-CoV-2 positive patients.

In SARS-CoV-2 infection, the role of the gut microbiome has been studied more widely; it has already been described that *Bacteroides dorei*, *Bacteroides thetaiotaomicron*, *Bacteroides massiliensis*, and *Bacteroides ovatus* downregulate expression of ACE2 receptors in the gut [15], and furthermore *Akkermansia muciniphila* and *Faecalibacterium prausnitzii* treatments appear to decrease the expression levels of cathepsin L [16]. The correlation between the abundance of several members of the genus *Streptococcus* and systemic inflammatory markers in SARS-CoV-2 infection was observed in the gut microbiome as well [17].

To the best of our knowledge, our study is the first to examine the role of human nasopharyngeal microbiota in regulating the expression of ACE2, TMPRSS2, and cathepsin L the entry gates of the SARS-CoV-2 virus. Based on our results, *C. accolens* and some defined *C. propinquum* strains significantly reduced the expression of ACE2, TMPRSS2, and cathepsin L, all of which are essential for adherence of the virus to cells [18]. The ACE2 receptor is essential for the attachment of the SARS-CoV-2 virus to the cell surface, but it depends on the SARS-CoV-2 mutants whether it enters the cell by membrane fusion with the TMPRSS2 receptor, for example, beta variant, or by endocytosis with the cathepsin L, for example, omicron variant. *C. accolens* inhibits both cell entry pathways, so its protective effect is mutant-independent. Furthermore, compounds produced by some *Corynebacterium* strains weakened the strength of binding between the SARS-CoV-2 spike protein and ACE2 receptor.

TAG lipase LipS1 produced by *C. accolens* is likely to be extracellular [19] and we assume that it could hydrolyze the coronavirus envelope as well*.* Furthermore, TAG lipase LipS1 releases oleic acid from oleoylethanolamide through triolein hydrolysis, and thus inhibits the release of proinflammatory cytokines induced by SARS-CoV-2 [20].

*Corynebacterium* and *Streptococcus* strains are often involved in the formation of the normal nasopharyngeal microbiota and *Corynebacterium* is associated with a healthy state [21, 22]. Our results indicate that individual susceptibility to SARS-CoV-2 infection is associated with differences in the overall nasopharyngeal bacterial community structure. It appears that *Corynebacterium* spp. have a major role in the prevention of infection and these bacteria decrease the infection rate via multiple mechanisms.

**Fig. 3:**
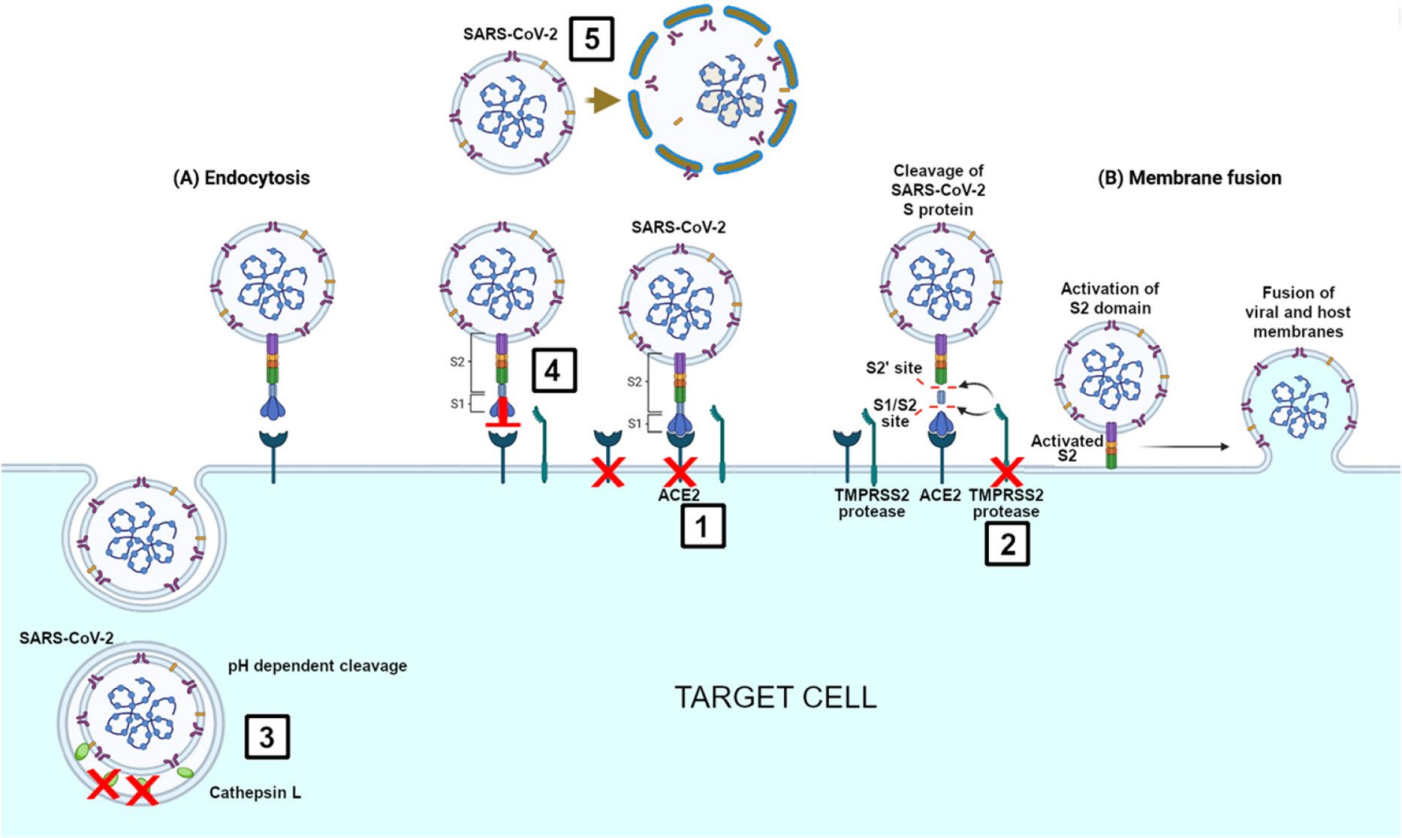
The different protective mechanisms of *Corynebacterium accolens* against SARS-CoV-2 infection. 1. By downregulation of ACE2 receptors on the target host cell decreasing the ACE2-SARS-CoV-2 binding. 2. By downregulation of TMPRSS2 receptors on the target host cell inhibiting the virus SARS-CoV-2 entry by membrane fusion. 3. By downregulation of cathepsin L in the endosome inhibiting the virus SARS-CoV-2 entry by endocytosis. 4. Inhibiting the binding strength between the S1 protein and ACE2 receptor. 5. Destabilizing the envelope of SARS-CoV-2 by TAG lipase LipS1 of *C. accolens*

In conclusion, presence of *Corynebacterium* spp. in the nasopharyngeal microbiota, especially *C. accolens* strains, could reduce the individual susceptibility to SARS-CoV-2 infection by several mechanisms including downregulation (1) the ACE2 and (2) the TMPRSS2 receptors of SARS-CoV-2; and SARS-CoV-2 endocytosis by (3) downregulation cathepsin L expression; (4) through the inhibition of S1 protein—ACE2 receptor binding; and (5) through TAG lipase production. These mechanisms together act by inhibiting the binding and the entry of SARS-CoV-2 to the host cell and by acting on the lipid envelope of the SARS-CoV-2 virus (Fig. 3).

These features definitely provide an advantage to the host against the virus and correlate well with the finding of the greater relative abundance of nasal *Corynebacterium *spp. in individuals who remain free of infection.

A subset of *Corynebacterium* spp. as a probiotic in the nasal cavity may be a protective gatekeeper for SARS-CoV-2 and a potential candidate to prevent infection or asymptomatic transmission of coronavirus and other lipid-enveloped respiratory viruses.

A limitation of the current study is the low number of patients. However, here we presented a unique study investigating unvaccinated close contacts individuals, persons involved in the same viral exposure.
